# Target of rapamycin (TOR) regulates the response to low nitrogen stress via autophagy and hormone pathways in *Malus hupehensis*

**DOI:** 10.1093/hr/uhac143

**Published:** 2022-06-27

**Authors:** Danyang Li, Yuduan Ding, Li Cheng, Xiaoli Zhang, Siyuan Cheng, Ying Ye, Yongchen Gao, Ying Qin, Zhu Liu, Cuiying Li, Fengwang Ma, Xiaoqing Gong

**Affiliations:** State Key Laboratory of Crop Stress Biology for Arid Areas/Shaanxi Key Laboratory of Apple, College of Horticulture, Northwest A&F University, Yangling 712100, Shaanxi, China; State Key Laboratory of Crop Stress Biology for Arid Areas/Shaanxi Key Laboratory of Apple, College of Horticulture, Northwest A&F University, Yangling 712100, Shaanxi, China; State Key Laboratory of Crop Stress Biology for Arid Areas/Shaanxi Key Laboratory of Apple, College of Horticulture, Northwest A&F University, Yangling 712100, Shaanxi, China; State Key Laboratory of Crop Stress Biology for Arid Areas/Shaanxi Key Laboratory of Apple, College of Horticulture, Northwest A&F University, Yangling 712100, Shaanxi, China; State Key Laboratory of Crop Stress Biology for Arid Areas/Shaanxi Key Laboratory of Apple, College of Horticulture, Northwest A&F University, Yangling 712100, Shaanxi, China; State Key Laboratory of Crop Stress Biology for Arid Areas/Shaanxi Key Laboratory of Apple, College of Horticulture, Northwest A&F University, Yangling 712100, Shaanxi, China; State Key Laboratory of Crop Stress Biology for Arid Areas/Shaanxi Key Laboratory of Apple, College of Horticulture, Northwest A&F University, Yangling 712100, Shaanxi, China; State Key Laboratory of Crop Stress Biology for Arid Areas/Shaanxi Key Laboratory of Apple, College of Horticulture, Northwest A&F University, Yangling 712100, Shaanxi, China; State Key Laboratory of Crop Stress Biology for Arid Areas/Shaanxi Key Laboratory of Apple, College of Horticulture, Northwest A&F University, Yangling 712100, Shaanxi, China; State Key Laboratory of Crop Stress Biology for Arid Areas/Shaanxi Key Laboratory of Apple, College of Horticulture, Northwest A&F University, Yangling 712100, Shaanxi, China; State Key Laboratory of Crop Stress Biology for Arid Areas/Shaanxi Key Laboratory of Apple, College of Horticulture, Northwest A&F University, Yangling 712100, Shaanxi, China; State Key Laboratory of Crop Stress Biology for Arid Areas/Shaanxi Key Laboratory of Apple, College of Horticulture, Northwest A&F University, Yangling 712100, Shaanxi, China

## Abstract

Target of rapamycin (TOR) is a highly conserved master regulator in eukaryotes; it regulates cell proliferation and growth by integrating different signals. However, little is known about the function of TOR in perennial woody plants. Different concentrations of AZD8055 (an inhibitor of TOR) were used in this study to investigate the role of TOR in the response to low nitrogen (N) stress in the wild apple species *Malus hupehensis*. Low N stress inhibited the growth of *M. hupehensis* plants, and 1 μM AZD alleviated this effect. Plants supplied with 1 μM AZD had higher photosynthetic capacity, which promoted the accumulation of biomass, as well as higher contents of N and anthocyanins and lower content of starch. Exogenous application of 1 μM AZD also promoted the development of the root system. Plants supplied with at least 5 μM AZD displayed early leaf senescence. RNA-seq analysis indicated that TOR altered the expression of genes related to the low N stress response, such as genes involved in photosystem, starch metabolism, autophagy, and hormone metabolism. Further analysis revealed altered autophagy in plants supplied with AZD under low N stress; the metabolism of plant hormones also changed following AZD supplementation. In sum, our findings revealed that appropriate inhibition of TOR activated autophagy and jasmonic acid signaling in *M. hupehensis*, which allowed plants to cope with low N stress. Severe TOR inhibition resulted in the excessive accumulation of salicylic acid, which probably led to programmed cell death in *M. hupehensis*.

## Introduction

Apple is one of the most economically important fruits in the world [1]; it is also one of the main fruit tree species in northern China, such as the Bohai Bay area and the Loess Plateau. Apple orchards mainly occur in barren areas, and nutrients in the soil in such areas are often depleted. Nitrogen (N) is an essential nutrient that plays a key role in plant growth, development, and production; it is an integral element of the basic components of life, such as proteins and nucleic acids [2]. However, >98% of N in soil is organic N, which plants are not able to use efficiently [3]. The use of chemical N fertilizers in orchards has increased to promote the development of apple trees and the production of apple fruits. But the application rate in most orchards exceeds demand and results in negative consequences, such as low N utilization efficiency, which exacerbates shortages of N in apple trees. The part of N not absorbed would eventually leach into rivers or evaporate into the air, accelerating environmental degradation [4, 5]. Achieving high yield under N-limited conditions and reducing N fertilizer input are major challenges in apple production and require urgent attention.

N is an essential element for the synthesis of nucleic acids, proteins, lipids, and hormones. N deficiency affects cell division and differentiation, as well as many other physiological and biochemical processes, and alters plant morphology [6]. In the absence of N, chlorophyll synthesis is impaired; the subsequent decline in the rate of photosynthesis results in lower biomass production [7]. Plants have evolved several mechanisms to cope with N starvation stress. They can modify the architecture of their roots, especially the deep roots, and promote the growth of lateral roots to enhance the efficiency of N absorption from the soil [8]. The transport of N from roots to shoots and N utilization also change in response to low N stress [9]. These processes involve a complex signaling network consisting of different regulators, like plant hormones. For example, auxins have been shown to be involved in the response of plants to low N stress. They enhance N absorption from soil by promoting root elongation and lateral root formation [10]. Cytokinins are signals of N saturation in plants [11]. Abscisic acid (ABA) inhibits the expression of *ABI2*, which is an enhancer that promotes N uptake by regulating NPF6.3 (also known as NRT1.1) [12]. Ethylene and salicylic acid (SA) also function in the response of plants to low N stress [13, 14]. The jasmonic acid (JA) signal could mediate large-scale systemic changes in N assimilation [15].

Target of rapamycin (TOR) is a serine/threonine protein kinase that is highly conserved in eukaryotes; it integrates nutrient, light, hormone, stress, and energy signals to regulate various biological processes, thereby promoting cell proliferation and growth [16]. Nutrient uptake and assimilation are central to the life activities of plants; TOR is a central node involved in the control of cell growth and can regulate signaling networks by sensing nutrient signals such as carbon (C), N, sulfur (S), and phosphorus [16, 17]. It coordinates a variety of complex processes to regulate the C/N balance in aboveground and belowground organs. N can regulate the growth and development of apical and shoot tips by activating the ROP2–TOR pathway, and N depletion strongly inhibits TOR activity [18]. TOR inhibition induces autophagy in plants [19], which is a conserved degradation and recycling mechanism in eukaryotes, and is involved in plant development [20], nutrient recycling [21], and responses to stresses [22, 23]. When plants experience N starvation, autophagy degrades unwanted or damaged organelles and proteins to recycle N, and promotes the absorption and assimilation of N to increase N use efficiency [24, 25].

TOR is a key regulator of various biological processes in organisms in response to environmental stimuli. Rapamycin is often used in humans and yeast to characterize the biological function of TOR and pathways relevant to it. However, most terrestrial plants are insensitive to rapamycin and show embryonic lethality when *TOR* is mutated. Consequently, several ATP-competitive TOR-specific inhibitors have been developed and used in various studies, including AZD8055, Torin1, Torin2, and KU0063794, which can effectively inhibit TOR kinase activity and alter plant growth [26, 27]. Here, we studied the role of TOR in *Malus hupehensis* plants under low N stress. We used a hydroponic system to impose low N stress on *M. hupehensis* plants and used AZD8055 (hereafter referred to as AZD) to inhibit TOR kinase activity. The effect of TOR on the growth of *M. hupehensis* under low N conditions is discussed, as well as the underlying mechanism of the TOR signaling pathway. Our results provide new insights into the role of TOR in the response of plants to low N stress, especially in perennial woody plants.

## Results

### Effects of TOR inhibitor on the growth of *M. hupehensis* under low N stress

**Figure 1:**
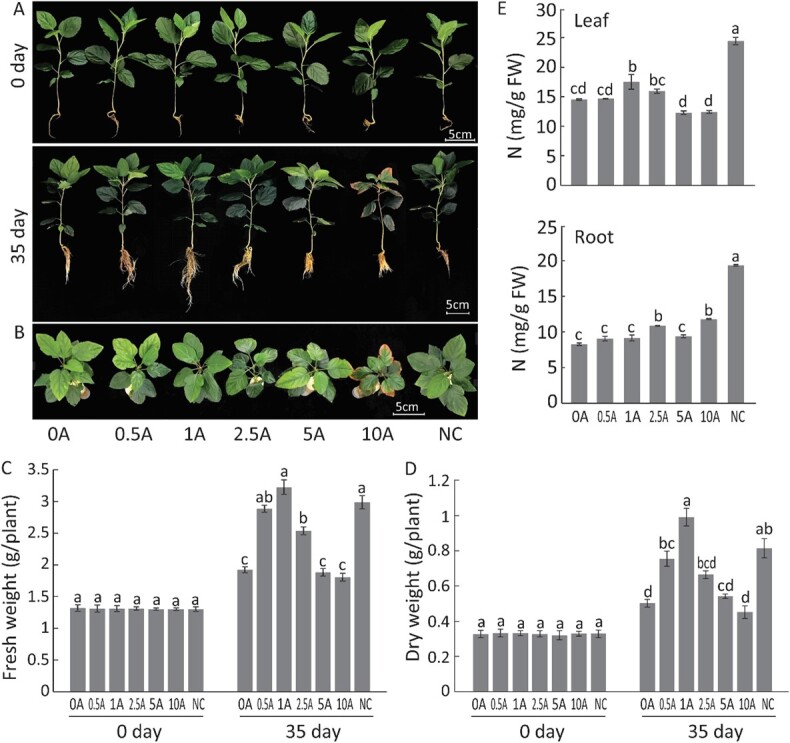
Response of *M. hupehensis* to low N stress with and without TOR inhibition. (A, B) Phenotypes of *M. hupehensis* under low N stress. (C, D) Average fresh and dry weights of plants measured before and after stress treatments. (E) Contents of N in roots and leaves in each treatment. Data are mean ± standard error of 10 replicate samples. Different lowercase letters indicate statistically significant differences between treatments according to Tukey’s multiple range test (*P* < .05). NC, plants without any treatment; 0A, low N stress treatment (0.2 mM N); 0.5A–10A, low N stress with 0.5 μM (0.5A), 1 μM (1A), 2.5 μM (2.5A), 5 μM (5A), and 10 μM (10A) AZD8055 treatments.

To evaluate the effects of TOR inhibition on the growth of *M. hupehensis* under low N stress, plants were cultured in a hydroponic system with normal (6 mM) and low N (0.2 mM) levels for 35 days and supplied with different concentrations of AZD. Compared with plants under control growth conditions (NC group), plants under low N stress had a stronger root system but chlorotic leaves. Among the six groups under low N stress (supplemented with 0, 0.5, 1.0, 2.5, 5, and 10 μM AZD and referred to as groups 0A, 0.5A, 1A, 2.5A, 5A, and 10A, respectively), the roots were longest in group 1A, and the leaves in this group were as green as those in the NC group. The roots were the second longest in the 0.5A and 2.5A groups; plants from the 5A and 10A groups were smaller and weaker, and leaf margin necrosis could be observed in them (Fig. 1A and B). After treatment, values for fresh and dry weight were much lower in the 0A group than those in the NC group, and were highest in the 1A group among the six low N groups, followed by the 0.5A group, in which values for fresh and dry weights were much higher than those in the other three groups (2.5A, 5A, and 10A) (Fig. 1C and D). In addition, the N content of the leaves and roots in the low N groups was much lower than that in the NC group at the end of the treatment period. However, the exogenous application of AZD increased the N content when it was applied at a suitable concentration (Fig. 1E).

Low N promoted the growth of roots (0A versus NC), and values for root length, root surface area, average root diameter, root volume, and numbers of tips and forks were higher (Fig. 1A, Table 1). The root architecture was also altered under TOR inhibition when different concentrations of AZD were applied in the hydroponic system. Values for all the root parameters increased with low concentrations of AZD and decreased with high concentrations of AZD (Table 1). These results indicated that low N conditions suppressed the growth of *M. hupehensis* plants, whereas the application of 1 μM AZD effectively alleviated the adverse effect of low N stress on *M. hupehensis*.

**Table 1:** Effects of TOR inhibition on root growth of *Malus hupehensis* plants under low N stress

	**Length (cm)**	**Surface area (cm** ^**2**^ **)**	**Average diameter (mm)**	**Root volume (cm** ^**3**^ **)**	**No. of tips**	**No. of forks**
0A	220.99 ± 22.90^b^ (100%)	34.68 ± 2.74^c^ (100%)	0.5301 ± 0.02^bc^ (100%)	0.3864 ± 0.02^cd^ (100%)	419.2 ± 29.44^abcd^ (100%)	1988 ± 158^bc^ (100%)
0.5A	326.07 ± 22.77^a^ (147%)	53.69 ± 3.12^a^ (154%)	0.5020 ± 0.01^c^ (94%)	0.7112 ± 0.04^ab^ (184%)	544.6 ± 90.89^ab^ (129%)	3273.8 ± 259^a^ (164%)
1A	356.08 ± 33.59^a^ (161%)	64.00 ± 5.86^a^ (184%)	0.5533 ± 0.01^bc^ (104%)	0.8566 ± 0.06^a^ (221%)	585.6 ± 35.83^a^ (139%)	3254.6 ± 369^a^ (163%)
2.5A	273.84 ± 12.05^ab^ (123%)	50.38 ± 2.14^ab^ (145%)	0.5777 ± 0.00^bc^ (108%)	0.7422 ± 0.05^a^ (192%)	452.8 ± 18.53^abc^ (108%)	2406.6 ± 189^ab^ (121%)
5A	190.93 ± 10.67^bc^ (86%)	38.39 ± 1.35^bc^ (110%)	0.7247 ± 0.02^a^ (136%)	0.6694 ± 0.04^ab^ (173%)	374.8 ± 30.10^bcd^ (89%)	1870.8 ± 47^bc^ (94%)
10A	192.86 ± 11.60^bc^ (87%)	36.22 ± 2.96^c^ (104%)	0.5721 ± 0.02^b^ (107%)	0.5350 ± 0.04^bc^ (138%)	305.8 ± 8.71 ^cd^ (72%)	2046 ± 253^bc^ (102%)
NC	113.40 ± 5.43^c^	18.21 ± 0.95^d^	0.4954 ± 0.00^c^	0.2450 ± 0.01^d^	256.8 ± 31.37^d^	1140 ± 120^c^

The number of tips and forks is a measure of the branchiness of the fine root system; a higher number of tips and forks means easier uptake of water and nutrients through the fine roots. All data are mean ± standard error of 10 plants. Percentages in parentheses after the standard error are for data in 0.5A, 1A, 2.5A, 5A, and 10A groups versus the 0A group. Different lowercase letters indicate statistically significant differences between treatments according to Tukey’s multiple range test (*P* < .05). NC, plants without any treatment; 0A, low N stress treatment (0.2 mM N); 0.5A–10A, low N stress with 0.5 μM (0.5A), 1 μM (1A), 2.5 μM (2.5A), 5 μM (5A), and 10 μM (10A) AZD8055 treatments.

### Effects of TOR inhibitor on the photosynthetic capacity of *M. hupehensis* under low N stress

**Figure 2:**
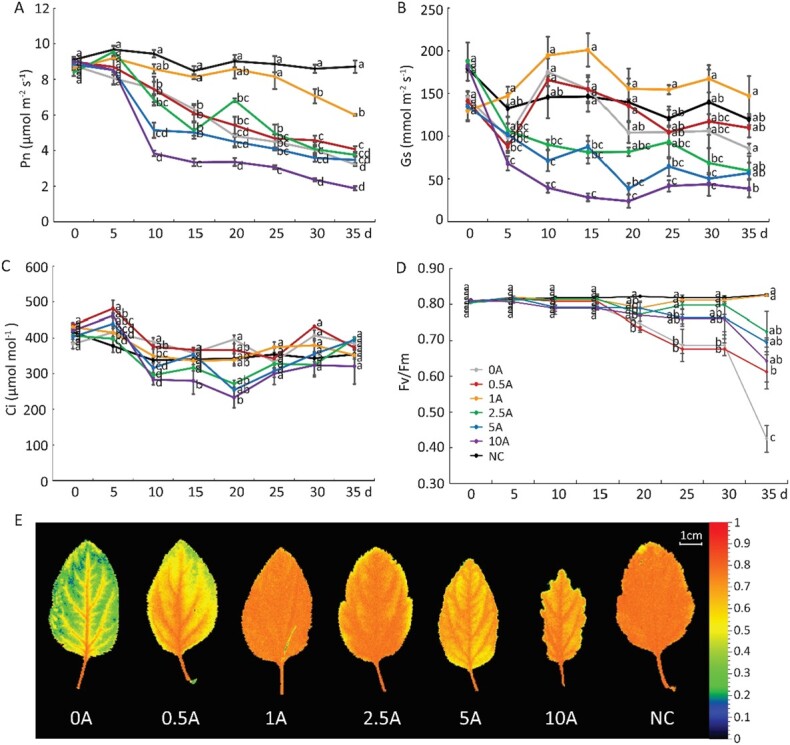
Effects of TOR inhibition on the photosynthetic capacity of *M. hupehensis* plants under low N stress. (A) Net photosynthesis (Pn). (B) Stomatal conductance (Gs). (C) Intercellular CO_2_ concentration (Ci). (D) Maximum potential PSII efficiency (Fv/Fm). (E) Chlorophyll fluorescence images of plants in different treatments. Data are mean ± standard error of 10 replicate samples. Different lowercase letters indicate statistically significant differences between treatments according to Tukey’s multiple range test (*P* < .05). NC, plants with no treatment; 0A, low N stress treatment (0.2 mM N); 0.5A–10A: low N stress with 0.5 μM (0.5A), 1 μM (1A), 2.5 μM (2.5A), 5 μM (5A), and 10 μM (10A) AZD8055 treatments.

Inhibition of TOR activity changed the biomass accumulation of plants under low N conditions; thus, we evaluated the photosynthetic capacity of plants. We made continuous measurements of the gas exchange parameters for all groups and found no difference in the net photosynthetic rate (Pn), stomatal conductance (Gs), or intercellular CO_2_ concentration (Ci) among them at the beginning of the treatments. Pn decreased in response to low N stress, and the differences among the seven groups became more pronounced over time. Under low N stress, plants supplied with a relatively low concentration of AZD had higher Pn values (e.g. 0.5A and 1A versus 0A); by contrast, plants supplied with a relatively high concentration of AZD had lower Pn values (e.g. 5A and 10A versus 0A) over time. At the end of the low N treatments, the lowest Pn value was found for the 10A group and the highest Pn value was found for the 1A group (Fig. 2A). Gs was much higher in the 1A group than in the other six groups, and it decreased over time in most groups (Fig. 2B). Ci fluctuated almost in the same pattern over time in all seven groups (Fig. 2C).

In addition, we compared the maximum photochemical efficiency of photosystem II (PSII) (Fv/Fm) among the seven groups. The Fv/Fm value was ~0.8 in the NC group. After treatment with low N for 35 days, the value of Fv/Fm gradually decreased to ~0.4 in the 0A group, and the exogenous application of AZD reversed this effect. The Fv/Fm value first increased and then decreased as the AZD concentration increased, and the highest value was observed in the 1A group, in which it was nearly the same as in the NC group (Fig. 2D and E). These data indicated that the reduction in the photosynthetic capacity of plants under low N stress was mitigated by exogenous AZD supplementation, especially by 1 μM AZD.

### Differential effects of TOR inhibition on accumulation of starch and anthocyanins in *M. hupehensis* under low N stress

**Figure 3:**
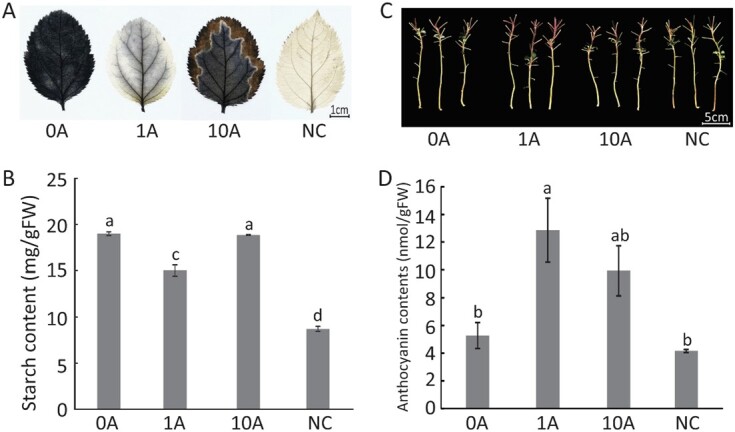
Effects of TOR inhibition on the accumulation of starch and anthocyanins in *M. hupehensis* plants under low N stress. (A) Staining of starch in leaves after low N treatment with exogenous AZD8055 for 35 days. (B) Starch content in leaves after low N treatment with exogenous AZD8055 for 35 days. (C) Red colors of stems after low N treatment with exogenous AZD8055 for 35 days. (D) Content of anthocyanins in plants after low N treatment with exogenous AZD8055 for 35 days. Data are mean ± standard error of five replicate samples. Different lowercase letters indicate statistically significant differences between treatments according to Tukey’s multiple range test (*P* < .05). NC, plants with no treatment; 0A, low N stress treatment (0.2 mM N); 1A and 10A, low N stress with 1 μM (1A) and 10 μM (10A) AZD8055 treatments.

We examined the effects of two extreme concentrations of AZD (1 μM for the positive role and 10 μM for the negative role) on the response of plants to N deficiency. C metabolism is tightly coupled to N metabolism, and starch is one of the two forms of carbohydrate stored in plant cells after C fixation [28]. In this study, we found that *M. hupehensis* plants accumulated large amounts of starch after low N treatment, as their leaves were shallow- or deep-stained blue. Among the four groups, the darkest blue was observed in the 0A group, followed by the 10A group. Leaves in the 1A group were stained light blue, and leaves in the NC group were almost white with a few blue flecks on the edge (Fig. 3A). This pattern was also consistent with the starch content observed in the four groups (Fig. 3B). These results demonstrated that the inhibition of TOR activity promoted the degradation of starch in N-deficient plants.

N deficiency modifies the secondary metabolism of plants and promotes the production of plant pigments such as anthocyanins, which could function as antioxidants in response to N deficiency [29]. Here, we found that the upper stems of plants turned red under low N conditions. The petioles of the upper leaves also turned red in response to low N stress. Among the four groups, deeper red was observed in the 1A group (Fig. 3C). We then measured the content of anthocyanins, which are responsible for red color of plants. Plants in the 1A group accumulated 12.86 nmol/g FW anthocyanins, which was approximately two and a half times higher compared with plants in the 0A group, and nearly three times higher compared with plants in the NC group (Fig. 3D), indicating that the beneficial role of appropriate TOR inhibition was partly linked to the accumulation of anthocyanins under low N stress.

### Differential effects of TOR inhibition on transcription level in *M. hupehensis* under low N stress

**Figure 4:**
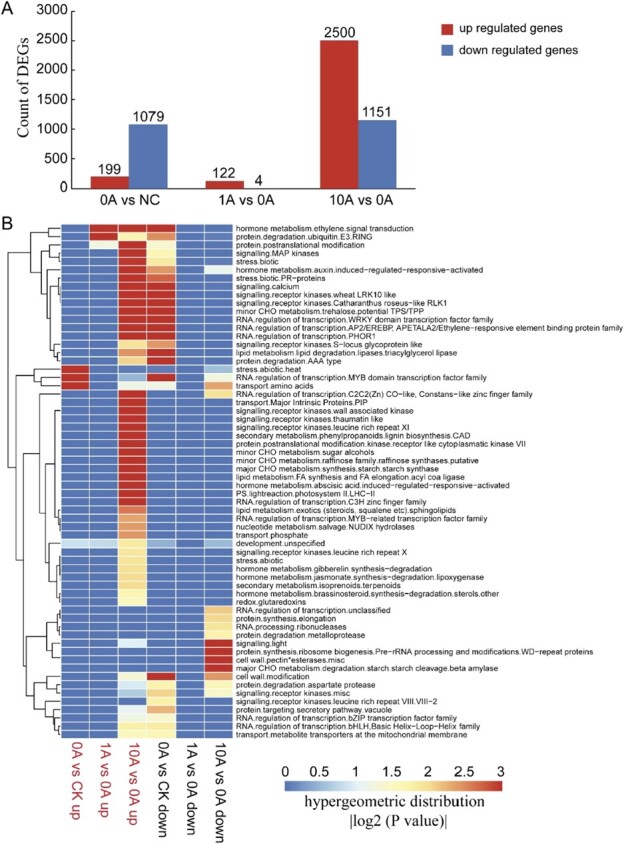
Effects of TOR inhibition on transcription in *M. hupehensis* plants under low N stress. (A) Numbers of up- and downregulated DEGs in different comparison groups. (B) MapMan enrichment analysis of DEGs in different comparison groups. The heat map shows the degree of enrichment [hypergeometric distribution *P*-value (|log2 (*P* value)|)]. NC, plants with no treatment; 0A, low N stress treatment (0.2 mM N); 1A and 10A, low N stress with 1 μM (1A) and 10 μM (10A) AZD8055 treatments.

Gene expression and its regulation were evaluated in response to TOR inhibition under low N stress. A total of 3923 differentially expressed genes (DEGs) were identified, which were clustered into eight groups according to their expression profiles (*P* < .05, log2 fold change >1) (Supplementary Data Fig. S1). Comparisons between groups 0A and NC, 1A and 0A, and 10A and 0A revealed 199, 122, and 2500 upregulated DEGs, respectively, and 1079, 4, and 1151 downregulated DEGs, respectively (Fig. 4A). Upregulated DEGs in the 1A versus 0A group were involved in hormone metabolism, protein degradation, and protein post-translation modification pathways, especially the former two. In the 10A versus 0A group, 2500 upregulated DEGs could be found in almost all identified clusters; moreover, downregulated DEGs were involved in protein synthesis, light signaling, major carbohydrate metabolism, and cell wall (Fig. 4B).

**Figure 5:**
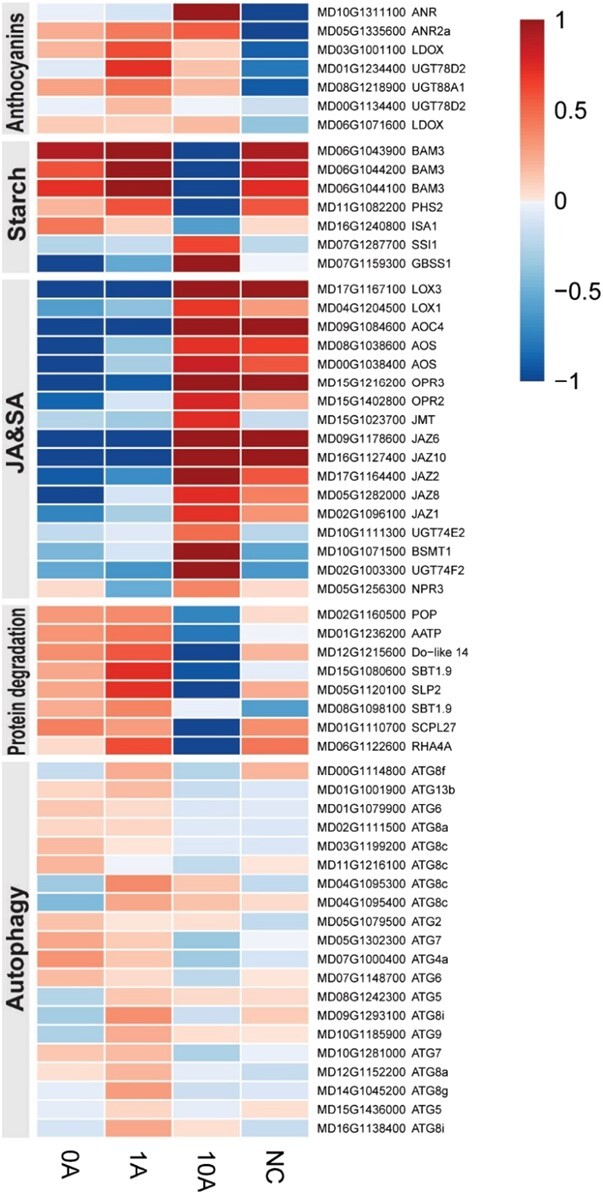
Heat map of some DEGs in the RNA-seq data. DEGs involved in anthocyanin, starch, and hormone metabolism, protein degradation, and autophagy are shown in the heat map. The normalized FPKM value is shown. NC, plants with no treatment; 0A, low N stress treatment (0.2 mM N); 1A and 10A, low N stress with 1 μM (1A) and 10 μM (10A) AZD8055 treatments.

The transcriptional profiles of DEGs involved in several biological processes were analyzed in different groups, including photosynthesis, N, starch, and anthocyanin metabolism, protein degradation, and plant hormone metabolism (Supplementary Data Table S2). Several DEGs are shown in Fig. 5. Genes involved in anthocyanin anabolism and starch degradation were highly expressed in the 1A group, and genes involved in anthocyanin reductase and starch synthesis were highly expressed in the 10A group, consistent with the content of anthocyanins and starch we detected in the four groups. Genes involved in protein degradation were grouped into two clusters; some of them were highly expressed in the 10A and NC groups, and the others oppositely were highly expressed in the 0A and 1A groups (Supplementary Data Table S2). However, the expression of all genes involved in protein degradation through autophagy peaked in the 1A group (Fig. 5). Moreover, genes involved in plant hormones were divided into four categories according to their functions, comprising synthesis, degradation, signal transduction, and induced-regulated-responsive-activated. Most of them were suppressed by low N stress (0A versus NC); among groups under low N stress, the expression of most genes was highest in the 10A group (Supplementary Data Table S2). For example, most genes involved in JA and SA synthesis and their related signal transduction pathways were highly expressed in the 10A group compared with the others (Fig. 5).

### Differential effects of TOR inhibition on autophagic activity in *M. hupehensis* under low N stress

**Figure 6:**
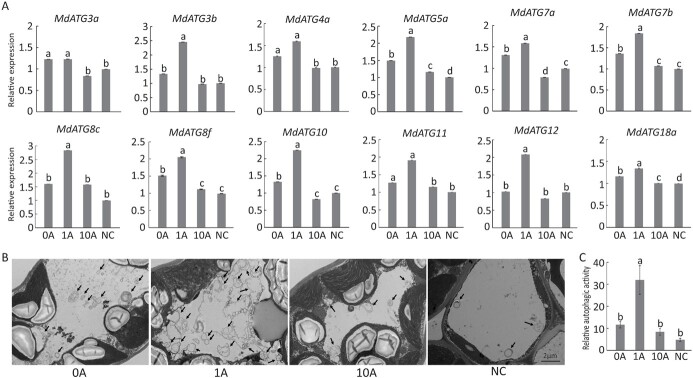
Effects of TOR inhibition on autophagy in *M. hupehensis* plants under low N stress. (A) Changes in the transcription level of apple autophagy-related genes under low N stress with different concentrations of AZD8055. *MDH* was used as internal control to normalize the relative expression levels of all *ATG* genes. Expression in the NC group was set to 1. Data are mean ± standard error of four replicate samples. (B) Representative transmission electron microscope images of autophagic structures in mesophyll cells from plants in the 0A, 1A, 10A, and NC groups. Autophagosomes are indicated by black arrows. Scale bars: 2 μm. (C) Relative autophagic activity of plants shown in (B). More than 10 cells were used to quantify structures; data are mean ± standard error of 10 replicate samples. Different lowercase letters indicate statistically significant differences between treatments according to Tukey’s multiple range test (*P* < .05).

TOR is the major negative regulator of autophagy [30]. We found that all genes involved in autophagy were upregulated in the 1A group in the RNA-seq data; thus, we performed several experiments to investigate how TOR affects autophagy in *M. hupehensis* under low N stress. We first analyzed the transcription levels of several autophagy-related genes (*ATG*s) [31, 32], and found that most of them were induced by low N stress (0A versus NC). The expression of *MdATG3b*, *-8c*, and -*12* was also increased, but not as much as other genes. In addition, application of 1 μM AZD increased the expression of all 12 candidate *MdATG*s. For example, the expression of *MdATG8c*, -*10*, and -*12* in the 1A group was nearly twice that in the NC group (Fig. 6A).

Transmission electron microscopy was used to observe autophagosome formation in leaves under low N stress (Fig. 6B and C). At the end of the experiments, plants in the NC group contained few autophagosome structures, whereas plants in the 0A group had a greater number of these structures. Three times as many autophagosomes and autophagic bodies could be observed in the 1A group (1A versus 0A). Overall, these data suggested that autophagic activity was significantly enhanced by appropriate inhibition of TOR activity in *M. hupehensis* under low N stress.

### Differential effects of TOR inhibition on hormone metabolism in *M. hupehensis* under low N stress

**Figure 7:**
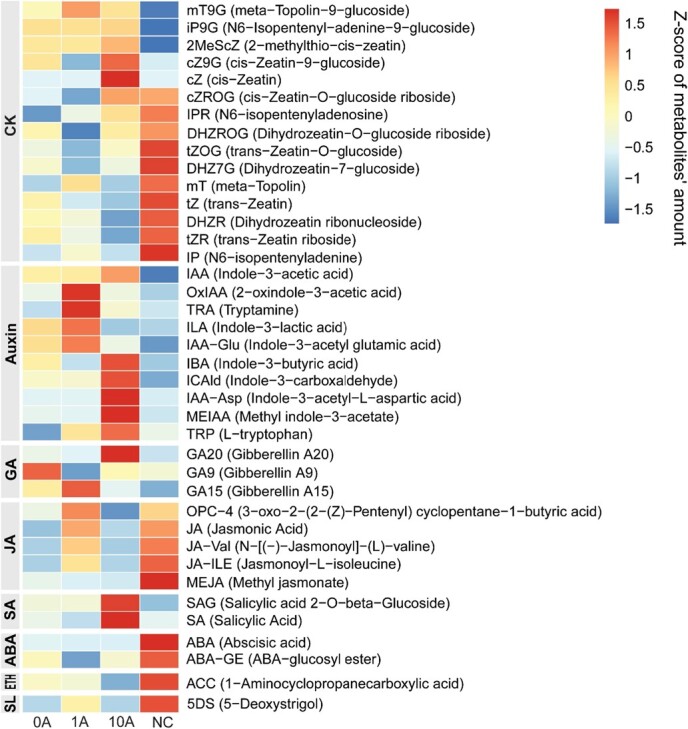
Representative plant hormones detected by UPLC and MS/MS in different treatments. The colors indicate the content (proportion) of each identified metabolite as determined by the average peak response area with *Z*-score normalization.

Several upregulated genes in the 1A versus 0A group were involved in hormone metabolism; they might be positively correlated with TOR inhibition under low N stress. Thus, we analyzed hormone metabolism in *M. hupehensis* plants. The accumulation of plant hormones changed dramatically in response to AZD, particularly in the 10A group; the total level of plant hormones was twice as high in the 10A group than in the NC group (Supplementary Data Fig. S2). We finally detected 39 different metabolites, which were assigned to cytokinin (15), auxin (10), gibberellin (3), JA (5), SA (2), ABA (2), ethylene (1), and strigolactone (1) (Supplementary Data Table S3, Supplementary Data Fig. S3). The accumulation of most metabolites in the cytokinin, JA, ABA, ethylene, and strigolactone groups was inhibited, whereas the accumulation of most metabolites in the auxin and SA groups was promoted by low N stress (0A versus NC). Differential inhibition of TOR activity further changed plant hormone metabolism under low N stress (Fig. 7).

The accumulation of metabolites in the cytokinin, auxin, and gibberellin groups changed irregularly upon TOR inhibition. In the auxin group, the content of six metabolites was highest in the 10A group, and the content of the other four was highest in the 1A group. The accumulation of the active forms indole-3-acetic acid (IAA) and l-tryptophan increased as the concentration of AZD increased. The accumulation of degradation products such as IAA-Glu, IAA-Asp, and OxIAA changed irregularly upon AZD application. The accumulation of ABA, ABA-glucosyl ester (ABA-GE), and 1-Aminocyclopropanecarboxylic acid (ACC) changed little following AZD application.

The accumulation of metabolites in the JA (5) and strigolactone groups (5-Deoxystrigol (5DS) only) under low N stress was promoted by 1 μM AZD and inhibited by 10 μM AZD, respectively. The excess accumulation of SA resulted in hypersensitivity reactions. The accumulation of SA and Salicylic acid 2-O-β-Glucoside (SAG), the most abundant metabolites we detected (Supplementary Data Fig. S2), peaked in the 10A group, which was consistent with the observed necrosis in the leaves.

In summary, these findings indicated that the positive effect of appropriate TOR inhibition on *M. hupehensis* under low N stress might be related to JA. IAA metabolism was antagonistic to TOR activity in leaves, and SA might cooperate with TOR in inducing necrosis in plants.

## Discussion

### Inhibition of TOR activity alters the response to low N stress in *M. hupehensis*

N is one of the main nutrients limiting plant growth and production, as it is the major element of most compounds in cells. N starvation prevents cells from synthesizing new proteins, nucleic acids, and other essential substances for plant growth, leading to growth retardation. N starvation also inhibits the synthesis of chlorophyll, reduces the photosynthetic capacity of plants, and results in yield decreases in plants [4, 7]. In this study we did not observe dwarf plants and the roots were stronger under low N stress; growth retardation was exhibited by the obvious reduction in the biomass of plants under low N stress (Fig. 1, Table 1).

Plants have evolved various mechanisms to ensure their growth and development under various environmental conditions, and the TOR signaling pathway is a conserved and central regulatory hub in these processes in all eukaryotes [33]. It senses various signals that affect plants, including nutrients, hormones, and stress signals [34, 35]. Inorganic N and amino acids can activate the TOR signaling pathway to promote development [36]. We also found that the TOR signaling pathway responded to low N stress, and there was a dosage effect between TOR inhibition and the low N stress response in *M. hupehensis*. When we applied AZD to *M. hupehensis* plants to inhibit TOR activity under low N stress, plants supplied with a low concentration of AZD (1 μM) accumulated more biomass and had stronger roots and higher photosynthetic efficiency; the opposite pattern was observed for plants supplied with a high concentration of AZD (10 μM) (Figs 1 and 2, Table 1). AZD at 1 μM alleviated the suppressive effect of low N stress on plants, whereas AZD intensified the suppressive effect beyond 5 μM, which confirmed that TOR plays a core role in regulating various biological processes in plants; the severe inhibition of its activity or its mutants results in the severe suppression of plant growth and can even induce lethal phenotypes.

### Appropriate inhibition of TOR activity promotes starch catabolism and anthocyanin anabolism in *M. hupehensis* to alleviate low N stress

**Figure 8:**
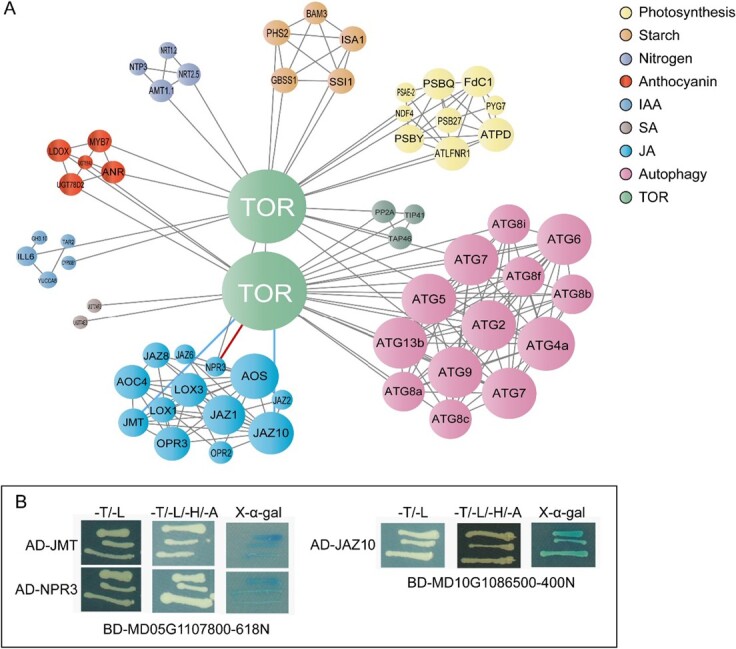
PPI analysis of DEGs. (A) PPI network. The protein interaction relationships of targeted DEGs were determined based on the genome-wide PPI network of apple (GDDH13). The predicted protein associations were determined from the STRING database, and the PPI was built based on experimental, text mining, database, and co-expression evidence. The size of the node is the parameter of the connectivity of the network. Multiple copies of identical genes are shown as a single node. For example, the large green circle at the bottom of the figure represents five sequences predicted as the *TOR* gene in the RNA-seq data, including *MD05G1107800*, *MD00G1031100*, *MD10G1086500*, *MD10G1112000*, and *MD15G1045400*. They coincided completely in the PPI network. The top small green circle represents novel.4829 in the RNA-seq data. (B) Interaction analysis between predicted TOR and proteins in the JA pathway. The 618 amino acids of the MD05G1107800 N-terminal and 400 amino acids of the MD10G1086500 N-terminal were used to construct BD vectors. JMT, JAZ10, and NPR3 were separately cloned into AD vectors. The recombined BD and AD vectors were then co-transformed into yeast and grew on different media. T, L, H, and A are short for Trp, Leu, His, and Ade. The line connecting TOR and NPR3 was colored red after the interaction was confirmed in yeast. The blue line connecting TOR and JMT and the blue line connecting TOR and JAZ10 were added to the PPI network according to the results in yeast.

C-containing compounds are the basic and abundant organic compounds in plant cells; most of them cannot be synthesized without N. N deficiency leads to excessive C in cells, resulting in surplus carbohydrates stored in chloroplasts as starch [9, 37]. This interplay between low N and starch accumulation also exists in *M. hupehensis*; we observed excessive accumulation of starch in leaves under low N stress (Fig. 3A). Generally, starch in chloroplasts is transitory; it is degraded into glucose when it enters C metabolism in plants. The excessive accumulation of starch in chloroplasts disrupts the distribution of C in plant organs, which leads to energy metabolism imbalance and eventually growth suppression [38]. TOR is a master regulator that regulates central energy metabolism, C partitioning, and growth in plants; inhibition of its activity results in the massive accumulation of storage lipids and starch [39, 40]. This probably explains the high amount of starch observed in plants in the 10A group, in which TOR activity was severely inhibited. However, when the inhibition of TOR activity was weak, as it was in the 1A group, the accumulation of starch decreased (Fig. 3A and B). Furthermore, we found that several genes involved in starch synthesis, such as *ISA1*, *SSI1*, and *GBSS1*, were highly expressed in the 10A and 0A groups, whereas genes involved in starch degradation, such as *BAM3* and *PHS2*, were highly expressed in the 1A group (Fig. 5). TOR can directly interact with these genes in the protein–protein interaction (PPI) network; it probably alters their expression to regulate the accumulation of starch under low N stress (Fig. 8A).

In addition, when sufficient N for synthesizing amino acids is not available, the excess of C-containing compounds might result in the synthesis of anthocyanins, which are the most abundant secondary metabolites in plants; they can then contribute substantially to low N stress tolerance in plant species [24, 41, 42]. We found that 1 μM AZD promoted the accumulation of anthocyanins in *M. hupehensis* under low N stress; the upper stems appeared redder than those in other groups (Fig. 3C and D). Several genes involved in the synthesis of anthocyanins, such as *LDOX*, *UGT78D2*, and *UGT88A1*, were highly expressed in the 1A group (Fig. 5); these genes also directly interacted with TOR in the PPI network (Fig. 8A). However, the interaction between TOR and these genes, as well as the mechanism by which TOR regulates them, requires further experimental confirmation.

### Appropriate inhibition of TOR activity promotes autophagy in *M. hupehensis* to alleviate low N stress

Precise regulation of the spatiotemporal expression of TOR is critical for plant growth and development. Appropriate TOR inhibition is beneficial for improving N use efficiency under low N stress, but excessive reduction of TOR activity leads to accelerated aging and abnormal apical dominance [18, 43, 44]. Autophagy is an important and highly conserved process in plants adapting to nutrient conditions. Changes in autophagy affect the remobilization, absorption, and transport of nutrient elements, which alters the adaptability of plants to N starvation conditions [45]. For example, enhanced autophagy promotes N uptake and assimilation in apples under low N stress [24]. It also controls S metabolism in leaves and facilitates S remobilization to seeds in *Arabidopsis* [46]. Autophagy regulates multiple genes in cells, and TOR is a negative upstream regulator of autophagy in both yeast and animals; this relationship has also been demonstrated in plants sensing nutrients [19, 30]. We found that the expression of several *ATG* genes was induced by low N stress, along with the increased number of autophagosomes in cells, and appropriate inhibition of TOR activity (treated with 1 μM AZD) further promoted autophagy, as revealed by the higher expression of *ATG* genes and the greater number of autophagosomes (Fig. 6). TOR has been shown to inhibit autophagy at the post-translational level by over-phosphorylating ATG13, which prevents its association with ATG1; TOR inhibition under nutrient starvation fails to dampen autophagy and leads to low N tolerance in plants [47]. With the exception of ATG13, TOR likely alters the expression of other *ATG* genes to regulate autophagy in cells, as we observed a direct interaction between TOR and nearly all the ATGs detected in the RNA-seq data in the PPI network (Fig. 8A). Appropriate inhibition of TOR induced autophagy, which facilitated the adaptation of *M. hupehensis* to low N stress; however, severe inhibition of TOR (treatment with 10 μM AZD) failed to activate autophagy. Plants were sensitive to low N stress, and their growth was inhibited (Figs 1 and 6). SnRK1 is another central metabolic regulator, which antagonizes TOR in regulating autophagy under energy-deficient conditions [48]. SnRK1 activates autophagy via the TOR signaling pathway; it also regulates autophagy in a TOR-independent manner [49]. It might have been responsible for the suppression of autophagy in the 10A group; however, we did not detect changes in the expression of genes involved in the SnRK1 signaling pathway in the RNA-seq data. We found that the expression of *TAP46*, *TIP41*, and *PP2A*, which function downstream of TOR to positively regulate autophagy [33], was suppressed by 10 μM AZD; this might explain why autophagy decreased in the 10A group. Other regulators and signal pathways related to TOR and autophagy might also be responsible for this pattern; more work is needed to clarify these findings.

### TOR regulates the metabolism of jasmonic acid and salicylic acid in *M. hupehensis* under low N stress

Plant hormone signaling activates responses to environmental stresses. Their integration with the TOR signaling pathway is essential and occurs frequently in plant cells [50–53]. The accumulation of JAs was higher in plants supplied with 1 μM AZD under low N stress. Under strong inhibition of TOR, plants accumulated SA instead (Fig. 7). JA and SA are important defense hormones and regulate plant growth and development. Exogenous methyl jasmonate improves growth characteristics and increases the lateral root numbers of *M. hupehensis* [54]. It also affects photosynthesis in a dose-dependent manner in *Citrus* plants [55]. These positive roles of JA likely contribute to the better growth of *M. hupehensis* when supplied with 1 μM AZD under low N stress. Although the crosstalk between TOR and JA remains unclear, TOR negatively affects the expression of genes related to JA biosynthesis and signal transduction in *Arabidopsis* and cotton [56, 57]. It suppresses JA signaling under abiotic stress [50]. In this study, we demonstrated the interaction between TOR (N-terminal) and NPR3 (Fig. 8B), which is a negative regulator in the JA pathway and a positive regulator in the SA pathway [58], and TOR also interacted with Jasmonic acid carboxyl methyltransferase (JMT) and Jasmonate ZIM-domain protein 10 (JAZ10) (Fig. 8B). JAZ10 is also a negative regulator in JA signaling transduction, and JMT is involved in the synthesis of JA. In addition, we found that TOR regulates JA signaling via some bridges, such as MYBs and MPKs (Supplementary Data Fig. S4). The present results suggested that TOR regulates the JA signaling pathway both directly and indirectly. Additional studies are still needed to reveal the crosstalk between TOR and JA in *M. hupehensis* responding to low N stress.

SA acts as a developmental regulator at low concentrations; it stimulates systemic acquired resistance to regulate plant defense responses at high concentrations [59]. Excessive accumulation of SA leads to hypersensitive reactions, which promotes leaf senescence in plants, also a form of programmed cell death [60]. This process is characterized by the high expression of a specific set of senescence-associated genes (*SAG*s). In plants supplied with 10 μM AZD under low N stress, the expression of several *SAG*s, such as *SAG101*, *SAG20*, and *SAG21* (Supplementary Data Table S2), was high, and leaf margin necrosis was observed (Fig. 1); SA content was also higher than in the other groups (Fig. 7), indicating the occurrence of programmed cell death. In sum, stronger TOR inhibition in the 10A group led to the excessive accumulation of SA, which resulted in early senescence in *M. hupehensis* under low N stress. Like JA, TOR antagonizes the action of SA in the plant immune system [61]. In addition, TOR might directly regulate the SA pathway; it interacted with *UGT74E2* and *UGT74F2*, which are related to SA metabolism, in the PPI (Fig. 8A), and with NPR3, which is a positive regulator of SA signaling, in yeast (Fig. 8B).

## Conclusions

**Figure 9:**
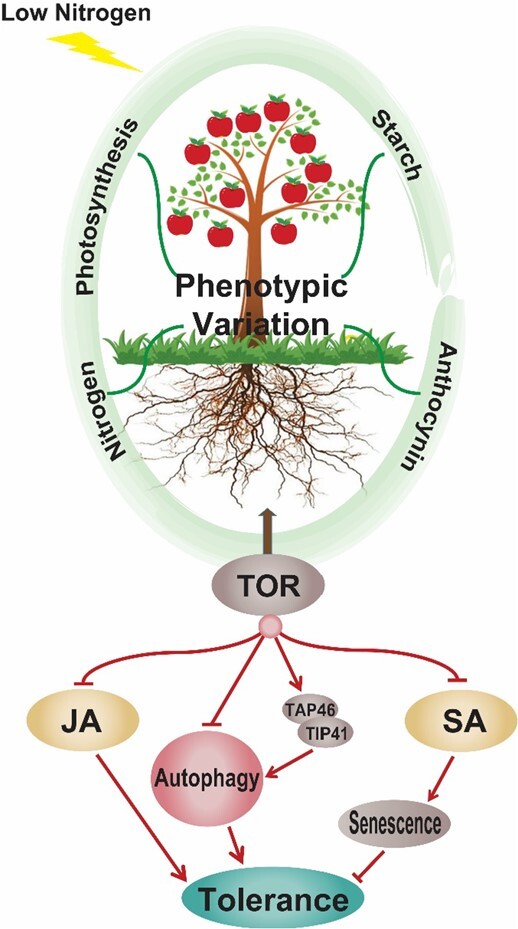
Proposed model of the regulatory function of TOR in response to low N stress in *M. hupehensis*. Under low N stress, TOR affects various physiological processes in *M. hupehensis* plants, such as photosynthesis and N, starch, and anthocyanin metabolism. This phenotypic variation is related to crosstalk between TOR and its downstream components. It negatively regulates JA and autophagy in plants to confer low N stress tolerance; TOR inhibition also cooperates with SA to cause programmed cell death in *M. hupehensis*.

In this study, we showed that low N repressed the growth of *M. hupehensis*. Application of AZD, an inhibitor of TOR, altered plant physiology profiles to mediate the response to stress (Fig. 9). In plants supplied with 1 μM AZD, biomass accumulation was greater, the root system was improved, the photosynthetic rate, N and anthocyanin contents were higher, and the starch content was lower. Further analysis revealed that autophagy was increased with the appropriate inhibition of TOR activity, which made plants recycle more nutrients under energy starvation. However, autophagy decreased when TOR was severely inhibited. TOR directly interacted with several ATGs in the PPI and altered their expression to regulate autophagy; it also indirectly regulated autophagy via *TAP46*. Finally, TOR altered plant hormone metabolism under low N stress. We suggest that TOR negatively regulates JA signaling in plants responding to low N stress; TOR inhibition also cooperates with SA to cause programmed cell death in *M. hupehensis*.

## Materials and methods

### Plant materials and growth

*M. hupehensis* was used because of its high degree of apomixis, which results in superior consistency during its development. The seeds were collected in Pingyi County (35°07′N, 117°25′E), Shandong Province, China. We cultivated the seedlings as described by Gong *et al.* [62]. Briefly, after stratification for 50 days at 4°C, germinated seeds were planted and cultured in a greenhouse. When six true leaves were fully expanded, seedlings were transferred to a hydroponic system with 1/2 Hoagland’s nutrient solution [63]. All the experiments were carried out at Northwest A & F University, Yang Ling (34°20′N, 108°24′E), China.

### TOR inhibitor application and low N treatment

To determine whether TOR plays a role in *M. hupehensis* under low N stress, different concentrations of the TOR inhibitor AZD were applied in the hydroponic system. All the seedlings were pre-cultured for 2 weeks, and uniform seedlings were selected and divided into a control group and a low N treatment group, with ~6 mM N and 0.2 mM N in 1/2 Hoagland nutrient solution, respectively. AZD was applied to the low N group at six different concentrations (0, 0.5, 1.0, 2.5, 5, and 10 μM), which were refreshed every 5 days along with the nutrient solution. Overall, there were two groups and seven different treatments (48 plants each) as follows: (i) normal conditions with 1/2 Hoagland nutrient solution (hereafter referred to as NC; the N level was ~6 mM); (ii) low N conditions with 0.2 mM N in 1/2 Hoagland nutrient solution, with 0, 0.5, 1.0, 2.5, 5, and 10 μM AZD supplementation separately; these six latter treatments were subsequently referred to as 0A, 0.5A, 1A, 2.5A, 5A, and 10A, respectively. The stress period ended after 35 days, and different parts of the selected plants were collected for measurements.

### Growth measurements

At the end of the experiment, 10 seedlings were randomly selected from each group to measure the growth parameters, including fresh and dry weights and root architecture according to Gong *et al.* [62]. Root architecture was first captured using an Epson V700 scanner (Epson, Japan), and root morphological indexes, including root length, surface area, average diameter, root volume, numbers of root tips and forks, were analyzed using the WinRHIZO Pro root analysis system (Regent Instruments, Quebec, Canada).

### Evaluation of photosynthetic efficiency and related parameters

The photosynthetic capacity of the seedlings was determined every 5 days at 9.00 a.m. during the study period using a CIRAS-3 portable photosynthesis system (PP Systems, USA), according to Jia *et al*. [32]. Ten individually mature and fully expanded leaves were measured for each treatment. The chlorophyll fluorescence was detected before and after the treatments according to Jia *et al*. [31], using an Open FluorCam FC 800-O imaging fluorometer (Photon Systems Instruments), and analyzed with Fluorcam7 software (PSI, Brno, Czech Republic).

### Determination of N, starch, and anthocyanin contents

After determining the dry weight, the samples (leaves and roots) were ground and sieved through a 60-mesh sieve. Samples (0.1 g) were then used to detect the N content in plants in different treatments, according to Sun *et al*. [24].

To dye leaf starch, the middle mature leaves of the plants were boiled in 95% absolute ethanol until the leaves turned white. They were then washed twice in ultrapure water and dyed in 5% Lugol’s solution (5% w/v I_2_ and 10% w/v KI) for 10 minutes, and then destained in water until a clear background was obtained. After that, leaves were photographed on a white background plate. Quantitative determination of starch and anthocyanin contents was carried out after extraction as described in a previous study [24].

### Detection of autophagy

Leaf autophagosomes were observed by transmission electron microscopy following the methods of Jia *et al.* [32]. At the end of each treatment, leaves were cut into small pieces, fixed in 4% glutaraldehyde solution in 0.1 M phosphate-buffered saline (pH 6.8), and then stored at 4°C for 12 hours. The autophagosomes were observed using an HT7700 transmission electron microscope (Hitachi, Japan). Quantitative PCR was conducted to analyze the transcript levels of *ATG* genes in different groups (Supplementary Data Table S1), according to Gong *et al*. [62]. There were four replicates for each sample.

### Measurement of plant hormone levels

Leaf samples were collected at 10 days for quantification of plant hormone levels in the different groups. After being ground into powder in liquid N, samples (50 mg) were mixed with internal standard and extracted in 1 mL of methanol/water/formic acid (15:4:1, v/v/v). The extracted supernatant was evaporated until dry with an N gas stream, redissolved in 100 μL of 80% methanol, and filtered through a 0.22-μm filter for liquid chromatography–tandem mass spectrometry (MS/MS) analysis. The data acquisition instrument system included ultraperformance liquid chromatography (UPLC) (ExionLC™ AD, USA, https://sciex.com.cn/) and MS/MS (TRAP^®^ 6500+, USA, https://sciex.com.cn/). There were three replicates for each sample.

### RNA-seq analysis

Leaf samples were collected at 10 days for transcriptome analysis following the RNA-seq methods of Hrdlickova *et al*. [64]. Total RNA was extracted, and the concentration and integrity of the RNA were assessed using a Qubit 2.0 fluorometer (Life Technologies, USA) and Agilent 2100 bioanalyzer (Agilent Technologies, USA), respectively. After removing rRNA, the rest of the sample was used to construct a library using the NEBNext^®^ Ultra™ RNA Library Prep Kit (NEB, USA) and diluted to 1.5 ng/μL. The insert size of the library was determined with the Agilent 2100 Bioanalyzer. The libraries were then sequenced on a Novaseq 6000 system. Raw data were filtered using fastp v0.19.3 (https://github.com/OpenGene/fastp); clean reads were retained for analysis, which were then mapped to the reference apple genome GDDH13 [65]. To estimate the expression of genes, the FPKM (fragments per kilobase of transcript per million reads) value of each gene was calculated based on the length of the gene and the number of reads mapped to each gene. Differential expression analysis between the two groups was performed using the DESeq2 package (v1.22.1). *P*-values were adjusted using the Benjamini–Hochberg procedure [66]. In addition, DEGs were annotated based on Gene Ontology (GO), Kyoto Encyclopedia of Genes Genomes (KEGG), and MapMan pathways [67]. The PPI network of DEGs was constructed using the STRING database [68].

### Yeast two-hybrid assay

The full-length sequences of three genes involved in JA (*NPR3*, *JMT*, and *JAZ10*) were cloned into the pGADT7 vectors, and the two predicted sequences of *TOR* (*MD05G1107800* and *MD10G1086500*) in the RNA-seq data were divided into several parts according to their predicted domains and then cloned into pGBKT7 vectors. The yeast two-hybrid assays were then performed as described in the Clontech protocol. All the primers are listed in Supplementary Data Table S1.

### Statistical analysis

Statistical analysis was performed using SPSS 22.0 software (IBM, Chicago, USA). Differences among means were determined by one-way ANOVA and Tukey’s multiple range test (*P* < .05).

## Acknowledgements

This work was supported by the National Key Research and Development Program of China (2018YFD1000305), the earmarked fund for the China Agricultural Research System (CARS-27), the Fundamental Research Funds for the Central Universities (2452019049), and the Natural Science Basic Research Program of Shaanxi (2022JQ-178).

## Author contributions

X.G. and Y.D. conceived the study; X.G. and D.L. designed the methodology; D.L., L.C., X.Z, S.C., Y.Y., and Y.Q. collected the data; D.L., Y.D., and X.G. analyzed the data and wrote the manuscript; F.M. and X.G. provided financial support; others prepared plant materials. All authors contributed critically to the drafts and gave final approval for publication.

## Data availability

All data included in this study are available in the article or supplementary materials, and the original RNA-seq data are available upon request by contact with the corresponding author (Xiaoqing Gong).

## Conflict of interest

The authors declare no conflict of interest.

## Supplementary data

Supplementary data is available at *Horticulture Research* online.

## Supplementary Material

Web_Material_uhac143

## References

[1] Migicovsky Z , Gardner KM, Money D et al. Genome to phenome mapping in apple using historical data. *Plant Genome*. 2016;9. 10.3835/plantgenome2015.11.0113 27898813

[2] Bloom AJ . The increasing importance of distinguishing among plant nitrogen sources. *Curr Opin Plant Biol*. 2015;25:10–6. 25899331 10.1016/j.pbi.2015.03.002

[3] Dechorgnat J , Nguyen CT, Armenguad P et al. From the soil to the seeds: the long journey of nitrate in plants. *J Exp Bot*. 2011;62:1349–59. 21193579 10.1093/jxb/erq409

[4] Kant S . Understanding nitrate uptake, signaling and remobilisation for improving plant nitrogen use efficiency. *Semin Cell Dev Biol*. 2018;74:89–96. 28838687 10.1016/j.semcdb.2017.08.034

[5] Toselli M , Baldi E, Cavani L et al. Soil-plant nitrogen pools in nectarine orchard in response to long-term compost application. *Sci Total Environ*. 2019;671:10–8. 30927723 10.1016/j.scitotenv.2019.03.241

[6] Chapin FS 3rd , Walter CH, Clarkson DT. Growth response of barley and tomato to nitrogen stress and its control by abscisic acid, water relations and photosynthesis. *Planta*. 1988;173:352–66. 24226542 10.1007/BF00401022

[7] Wen BB , Xiao W, Mu Q et al. How does nitrate regulate plant senescence? *Plant Physiol Bioch* . 2020;157:60–9. 10.1016/j.plaphy.2020.08.041 33091797

[8] Zhang HM , Jennings A, Barlow PW et al. Dual pathways for regulation of root branching by nitrate. *Proc Natl Acad Sci USA*. 1999;96:6529–34. 10339622 10.1073/pnas.96.11.6529 PMC26916

[9] Li G , Hu Q, Shi Y et al. Low nitrogen application enhances starch-metabolizing enzyme activity and improves accumulation and translocation of non-structural carbohydrates in rice stems. *Front Plant Sci*. 2018;9:1128. 30108604 10.3389/fpls.2018.01128 PMC6079283

[10] Hu SK , Zhang M, Yang Y et al. A novel insight into nitrogen and auxin signaling in lateral root formation in tea plant [*Camellia sinensis* (L.) O. Kuntze]. *BMC Plant Biol*. 2020;20:232. 32448156 10.1186/s12870-020-02448-7 PMC7247184

[11] Sakakibara H , Takei K, Hirose N. Interactions between nitrogen and cytokinin in the regulation of metabolism and development. *Trends Plant Sci*. 2006;11:440–8. 16899391 10.1016/j.tplants.2006.07.004

[12] Leran S , Edel KH, Pervent M et al. Nitrate sensing and uptake in *Arabidopsis* are enhanced by ABI2, a phosphatase inactivated by the stress hormone abscisic acid. *Sci Signal*. 2015;8:ra43. 25943353 10.1126/scisignal.aaa4829

[13] Conesa CM , Saez A, Navarro-Neila S et al. Alternative polyadenylation and salicylic acid modulate root responses to low nitrogen availability. *Plants (Basel)*. 2020;9:251. 10.3390/plants9020251 PMC7076428 32079121

[14] Khan MI , Trivellini A, Fatma M et al. Role of ethylene in responses of plants to nitrogen availability. *Front Plant Sci*. 2015;6:927. 26579172 10.3389/fpls.2015.00927 PMC4626634

[15] Wu XY , Ding C, Baerson SR et al. The roles of jasmonate signalling in nitrogen uptake and allocation in rice (*Oryza sativa* L.). *Plant Cell Environ*. 2019;42:659–72. 30251262 10.1111/pce.13451

[16] Wu Y , Shi L, Li L et al. Integration of nutrient, energy, light, and hormone signalling via TOR in plants. *J Exp Bot*. 2019;70:2227–38. 30715492 10.1093/jxb/erz028 PMC6463029

[17] Xiong Y , McCormack M, Li L et al. Glucose-TOR signalling reprograms the transcriptome and activates meristems. *Nature*. 2013;496:181–6. 23542588 10.1038/nature12030 PMC4140196

[18] Liu Y , Duan X, Zhao X et al. Diverse nitrogen signals activate convergent ROP2-TOR signaling in *Arabidopsis*. *Dev Cell*. 2021;56:1283–1295.e5. 33831352 10.1016/j.devcel.2021.03.022

[19] Liu Y , Bassham DC. TOR is a negative regulator of autophagy in *Arabidopsis thaliana*. *PLoS One*. 2010;5:e11883. 20686696 10.1371/journal.pone.0011883 PMC2912371

[20] Zhao P , Zhou XM, Zhao LL et al. Autophagy-mediated compartmental cytoplasmic deletion is essential for tobacco pollen germination and male fertility. *Autophagy*. 2020;16:2180–92. 31983274 10.1080/15548627.2020.1719722 PMC7751669

[21] Huang X , Zheng C, Liu F et al. Genetic analyses of the *Arabidopsis* ATG1 kinase complex reveal both kinase-dependent and independent autophagic routes during fixed-carbon starvation. *Plant Cell*. 2019;31:2973–95. 31615848 10.1105/tpc.19.00066 PMC6925010

[22] Bao Y , Song WM, Wang P et al. COST1 regulates autophagy to control plant drought tolerance. *Proc Natl Acad Sci USA*. 2020;117:7482–93. 32170020 10.1073/pnas.1918539117 PMC7132278

[23] Ismayil A , Yang M, Haxim Y et al. *Cotton leaf curl Multan virus* βC1 protein induces autophagy by disrupting the interaction of autophagy-related protein 3 with glyceraldehyde-3-phosphate dehydrogenases. *Plant Cell*. 2020;32:1124–35. 32051213 10.1105/tpc.19.00759 PMC7145496

[24] Sun X , Jia X, Huo L et al. MdATG18a overexpression improves tolerance to nitrogen deficiency and regulates anthocyanin accumulation through increased autophagy in transgenic apple. *Plant Cell Environ*. 2018;41:469–80. 29210078 10.1111/pce.13110

[25] Zhen X , Li X, Yu J et al. OsATG8c-mediated increased autophagy regulates the yield and nitrogen use efficiency in rice. *Int J Mol Sci*. 2019;20:4956. 10.3390/ijms20194956 PMC6801700 31597279

[26] Xiong F , Zhang R, Meng Z et al. Brassinosteriod insensitive 2 (BIN2) acts as a downstream effector of the target of rapamycin (TOR) signaling pathway to regulate photoautotrophic growth in *Arabidopsis*. *New Phytol*. 2017;213:233–49. 27479935 10.1111/nph.14118

[27] Montane MH , Menand B. ATP-competitive mTOR kinase inhibitors delay plant growth by triggering early differentiation of meristematic cells but no developmental patterning change. *J Exp Bot*. 2013;64:4361–74. 23963679 10.1093/jxb/ert242 PMC3808319

[28] Schlosser AJ , Martin JM, Hannah LC et al. The maize leaf starch mutation agps-m1 has diminished field growth and productivity. *Crop Sci*. 2012;52:700–6.

[29] Gould KS , McKelvie J, Markham KR. Do anthocyanins function as antioxidants in leaves? Imaging of H_2_O_2_ in red and green leaves after mechanical injury. *Plant Cell Environ*. 2002;25:1261–9.

[30] Neufeld TP . TOR-dependent control of autophagy: biting the hand that feeds. *Curr Opin Cell Biol*. 2010;22:157–68. 20006481 10.1016/j.ceb.2009.11.005 PMC2854204

[31] Jia X , Gong X, Jia X et al. Overexpression of MdATG8i enhances drought tolerance by alleviating oxidative damage and promoting water uptake in transgenic apple. *Int J Mol Sci*. 2021;22:5517. 34073724 10.3390/ijms22115517 PMC8197189

[32] Jia X , Mao K, Wang P et al. Overexpression of MdATG8i improves water use efficiency in transgenic apple by modulating photosynthesis, osmotic balance, and autophagic activity under moderate water deficit. *Hortic Res*. 2021;8:81. 33790273 10.1038/s41438-021-00521-2 PMC8012348

[33] Quilichini TD , Gao P, Pandey PK et al. A role for TOR signaling at every stage of plant life. *J Exp Bot*. 2019;70:2285–96. 30911763 10.1093/jxb/erz125

[34] Ingargiola C , Duarte GT, Robaglia C et al. The plant target of rapamycin: a conduc TOR of nutrition and metabolism in photosynthetic organisms. *Genes (Basel)*. 2020;11:1285. 10.3390/genes11111285 PMC7694126 33138108

[35] Liu Y , Xiong Y. Plant TOR signaling network: complexes, conservations and specificities. *J Integr Plant Biol*. 2021;64:342–70. 10.1111/jipb.13212 34964268

[36] Tulin F , Zhang ZZ, Wang ZY. Activation of TOR signaling by diverse nitrogen signals in plants. *Dev Cell*. 2021;56:1213–4. 33945780 10.1016/j.devcel.2021.04.014

[37] Robinson JM . Nitrogen limitation of soybean plants promoted increased starch levels and dark respiration rates in mature leaves. *Plant Physiol*. 1993;102:125–5.12231803

[38] Smith AM , Zeeman SC. Starch: a flexible, adaptable carbon store coupled to plant growth. *Annu Rev Plant Biol*. 2020;71:217–45. 32075407 10.1146/annurev-arplant-050718-100241

[39] Caldana C , Li Y, Leisse A et al. Systemic analysis of inducible target of rapamycin mutants reveal a general metabolic switch controlling growth in *Arabidopsis thaliana*. *Plant J*. 2013;73:897–909. 23173928 10.1111/tpj.12080

[40] Zhang Y , Persson S, Giavalisco P. Differential regulation of carbon partitioning by the central growth regulator target of rapamycin (TOR). *Mol Plant*. 2013;6:1731–3. 23761348 10.1093/mp/sst094

[41] Liang J , He JX. Protective role of anthocyanins in plants under low nitrogen stress. *Mol Cell Biol Res Commun*. 2018;498:946–53. 10.1016/j.bbrc.2018.03.087 29548824

[42] Meng JX , Gao Y, Han M et al. In vitro anthocyanin induction and metabolite analysis in *Malus spectabilis* leaves under low nitrogen conditions. *Hortic Plant J*. 2020;6:284–92.

[43] Deprost D , Yao L, Sormani R et al. The *Arabidopsis* TOR kinase links plant growth, yield, stress resistance and mRNA translation. *EMBO Rep*. 2007;8:864–70. 17721444 10.1038/sj.embor.7401043 PMC1973950

[44] Moreau M , Azzopardi M, Clément G et al. Mutations in the *Arabidopsis* homolog of LST8/GβL, a partner of the target of rapamycin kinase, impair plant growth, flowering, and metabolic adaptation to long days. *Plant Cell*. 2012;24:463–81. 22307851 10.1105/tpc.111.091306 PMC3315227

[45] Masclaux-Daubresse C , Chen QW, Have M. Regulation of nutrient recycling via autophagy. *Curr Opin Plant Biol*. 2017;39:8–17. 28528166 10.1016/j.pbi.2017.05.001

[46] Lornac A , Havé M, Chardon F et al. Autophagy controls sulphur metabolism in the rosette leaves of *Arabidopsis* and facilitates S remobilization to the seeds. *Cell*. 2020;9:332. 10.3390/cells9020332 PMC7073174 32023971

[47] Chen QW , Shinozaki D, Luo J et al. Autophagy and nutrients management in plants. *Cell*. 2019;8:1426. 10.3390/cells8111426 PMC6912637 31726766

[48] Margalha L , Confraria A, Baena-Gonzalez E. SnRK1 and TOR: modulating growth-defense trade-offs in plant stress responses. *J Exp Bot*. 2019;70:2261–74. 30793201 10.1093/jxb/erz066

[49] Soto-Burgos J , Bassham DC. SnRK1 activates autophagy via the TOR signaling pathway in *Arabidopsis thaliana*. *PLoS One*. 2017;12:e0182591. 28783755 10.1371/journal.pone.0182591 PMC5544219

[50] Rodriguez M , Parola R, Andreola S et al. TOR and SnRK1 signaling pathways in plant response to abiotic stresses: do they always act according to the “yin-yang” model? *Plant Sci* . 2019;288:110220. 31521220 10.1016/j.plantsci.2019.110220

[51] Deng K , Dong P, Wang W et al. The TOR pathway is involved in adventitious root formation in *Arabidopsis* and potato. *Front Plant Sci*. 2017;8:784. 28553309 10.3389/fpls.2017.00784 PMC5427086

[52] Sun XC , Chen H, Wang P et al. Low nitrogen induces root elongation via auxin-induced acid growth and auxin-regulated target of rapamycin (TOR) pathway in maize. *J Plant Physiol*. 2020;254:153281. 32971423 10.1016/j.jplph.2020.153281

[53] Zhuo F , Xiong F, Deng K et al. Target of rapamycin (TOR) negatively regulates ethylene signals in *Arabidopsis*. *Int J Mol Sci*. 2020;21:2680. 10.3390/ijms21082680 PMC7215648 32290539

[54] Mao JP , Niu C, Chen S et al. Effects of exogenous methyl-jasmonate on the morphology, hormone status, and gene expression of developing lateral roots in *Malus hupehensis*. *Sci Hortic*. 2021;289:110419.

[55] Qiu X , Xu Y, Xiong B et al. Effects of exogenous methyl jasmonate on the synthesis of endogenous jasmonates and the regulation of photosynthesis in citrus. *Physiol Plant*. 2020;170:398–414. 32691420 10.1111/ppl.13170

[56] Salem MA , Giavalisco P. Mutation in the *Arabidopsis* regulatory-associated protein TOR 1B (RAPTOR1B) leads to decreased jasmonates levels in leaf tissue. *Plant Signal Behav*. 2019;14:e1649567. 31382813 10.1080/15592324.2019.1649567 PMC6768200

[57] Song Y , Zhao G, Zhang X et al. The crosstalk between target of rapamycin (TOR) and jasmonic acid (JA) signaling existing in *Arabidopsis* and cotton. *Sci Rep*. 2017;7:45830. 28374843 10.1038/srep45830 PMC5379187

[58] Liu LJ , Sonbol FM, Huot B et al. Salicylic acid receptors activate jasmonic acid signalling through a non-canonical pathway to promote effector-triggered immunity. *Nat Commun*. 2016;7:13099. 27725643 10.1038/ncomms13099 PMC5062614

[59] Pasternak T , Groot EP, Kazantsev FV et al. Salicylic acid affects root meristem patterning via auxin distribution in a concentration-dependent manner. *Plant Physiol*. 2019;180:1725–39. 31036755 10.1104/pp.19.00130 PMC6752920

[60] Woo HR , Kim HJ, Lim PO et al. Leaf senescence: systems and dynamics aspects. *Annu Rev Plant Biol*. 2019;70:347–76. 30811218 10.1146/annurev-arplant-050718-095859

[61] De Vleesschauwer D , Filipe O, Hoffman G et al. Target of rapamycin signaling orchestrates growth-defense trade-offs in plants. *New Phytol*. 2018;217:305–19. 28905991 10.1111/nph.14785 PMC5711548

[62] Gong XQ , Shi ST, Dou FF et al. Exogenous melatonin alleviates alkaline stress in *Malus hupehensis* Rehd. by regulating the biosynthesis of polyamines. *Molecules*. 2017;22:1542. 10.3390/molecules22091542 PMC6151414 28902159

[63] Hoagland DR , Arnon DI. The water-culture method for growing plants without soil. *The College of Agriculture*. 1950; Available at: http://www.archive.org/stream/watercultureme3450hoag#page/n0/mode/2up.

[64] Hrdlickova R , Toloue M, Tian B. RNA-Seq methods for transcriptome analysis. *Wiley Interdiscip Rev RNA*. 2017;8:e1364. 10.1002/wrna.1364 PMC5717752 27198714

[65] Daccord N , Celton JM, Linsmith G et al. High-quality de novo assembly of the apple genome and methylome dynamics of early fruit development. *Nat Genet*. 2017;49:1099–106. 28581499 10.1038/ng.3886

[66] Benjamini Y , Hochberg Y. Controlling the false discovery rate – a practical and powerful approach to multiple testing. *J R Stat Soc Series B Stat Methodol*. 1995;57:289–300.

[67] Usadel B , Poree F, Nagel A et al. A guide to using MapMan to visualize and compare omics data in plants: a case study in the crop species, maize. *Plant Cell Environ*. 2009;32:1211–29. 19389052 10.1111/j.1365-3040.2009.01978.x

[68] Szklarczyk D , Gable AL, Nastou KC et al. The STRING database in 2021: customizable protein-protein networks, and functional characterization of user-uploaded gene/measurement sets. *Nucleic Acids Res*. 2021;49:D605–12. 33237311 10.1093/nar/gkaa1074 PMC7779004

