# Supplementary material for: Target of rapamycin (TOR) regulates the response to low nitrogen stress via autophagy and hormone pathways in Malus hupehensis
Source: Hortic Res. 2022 Jun 27;9:uhac143. doi: 10.1093/hr/uhac143 (PMC9437726; doi:10.1093/hr/uhac143)
Supplement: Web_Material_uhac143 [file web_material_uhac143.docx]

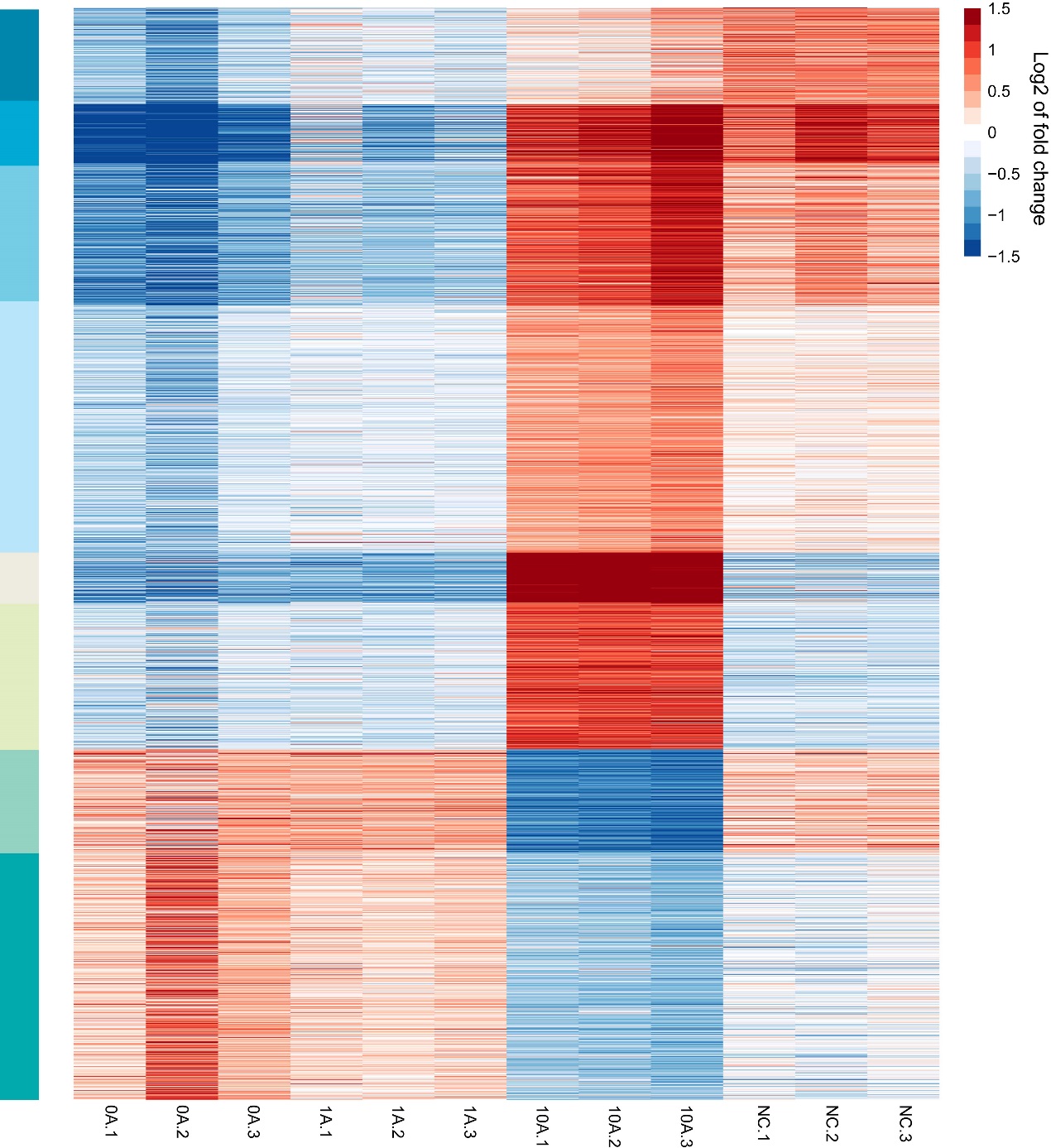


Supplemental Figure 1. Heat map of the identified differentially expressed genes in *Malus hupehensis* in different treatments. NC: Plants without any treatment; 0A: low N stress treatment (0.2 mM N); 1A and 10A: low N stress with 1 µM (1A) and 10 µM (10A) AZD8055 treatments.


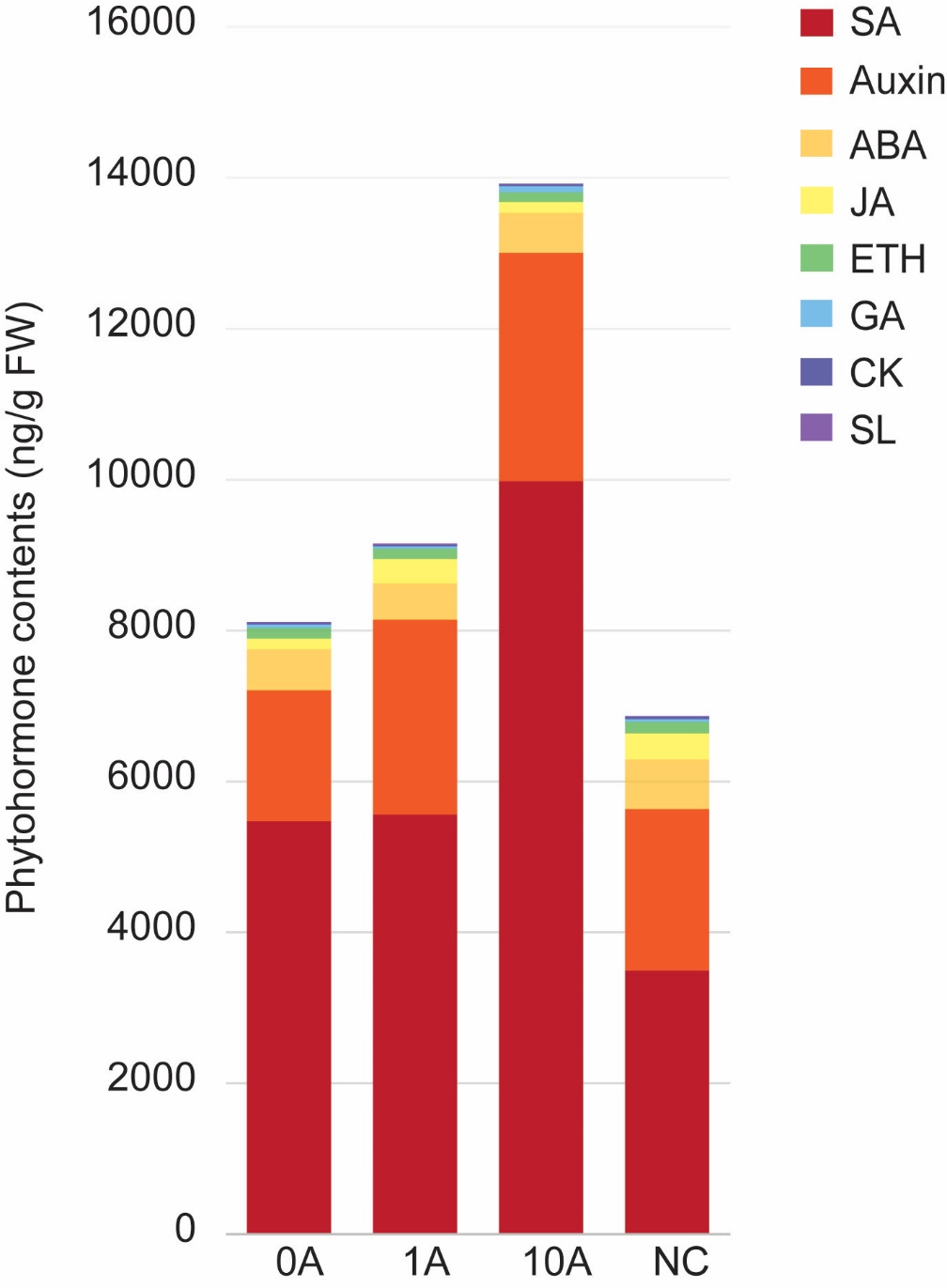


Supplemental Figure 2. The total content of all the detected hormone metabolites in different treatments. SA: salicylic acid, ABA: abscisic acid, JA: jasmonic acid, ETH: ethylene, GA: gibberellin, CK: cytokinin, SL: strigolactone. NC: Plants without any treatment; 0A: low N stress treatment (0.2 mM N); 1A and 10A: low N stress with 1 µM (1A) and 10 µM (10A) AZD8055 treatments.


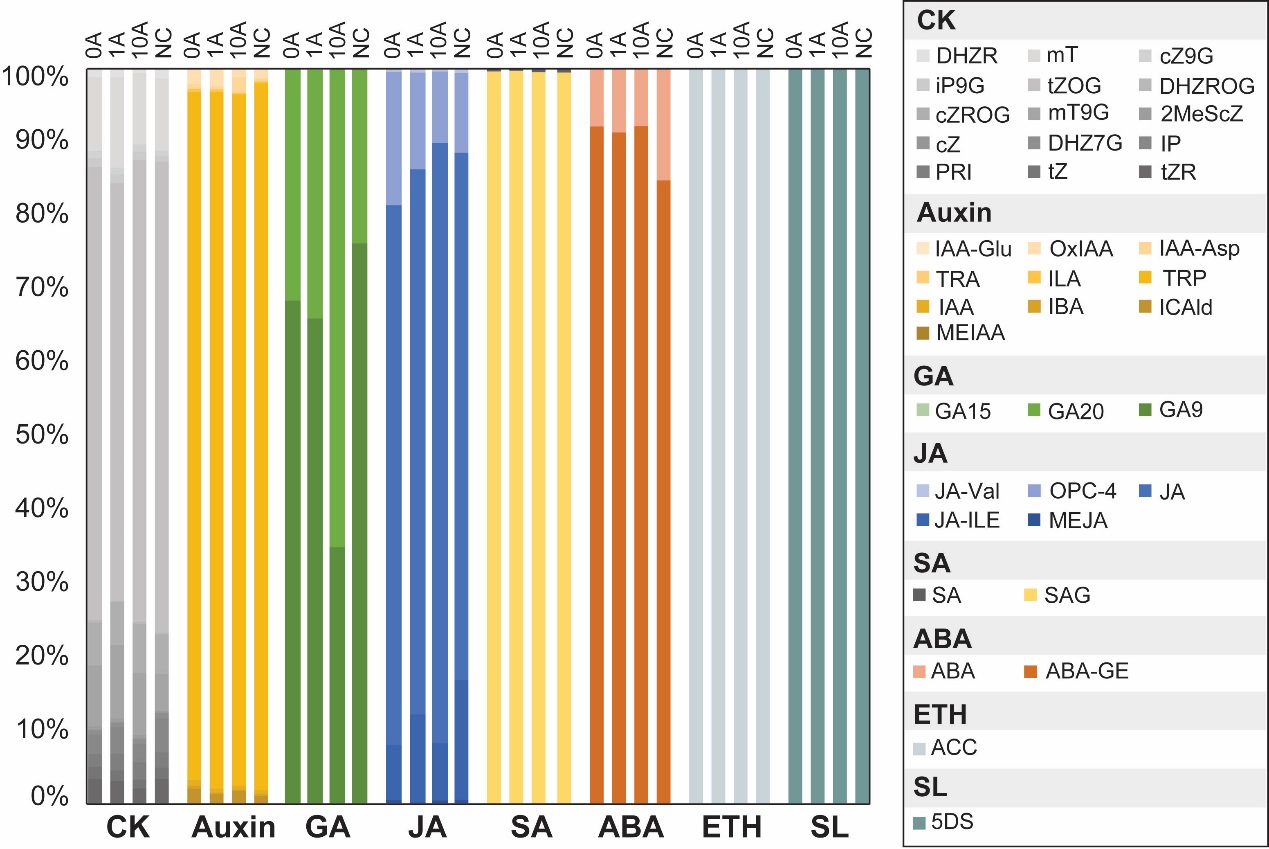


Supplemental Figure 3. Proportion of every detected metabolite in each hormone types in different treatments. SA: salicylic acid, ABA: abscisic acid, JA: jasmonic acid, ETH: ethylene, GA: gibberellin, CK: cytokinin, SL: strigolactone. The full names of each metabolite can be found in supplemental Table 2 or Figure 7. NC: Plants without any treatment; 0A: low N stress treatment (0.2 mM N); 1A and 10A: low N stress with 1 µM (1A) and 10 µM (10A) AZD8055 treatments.


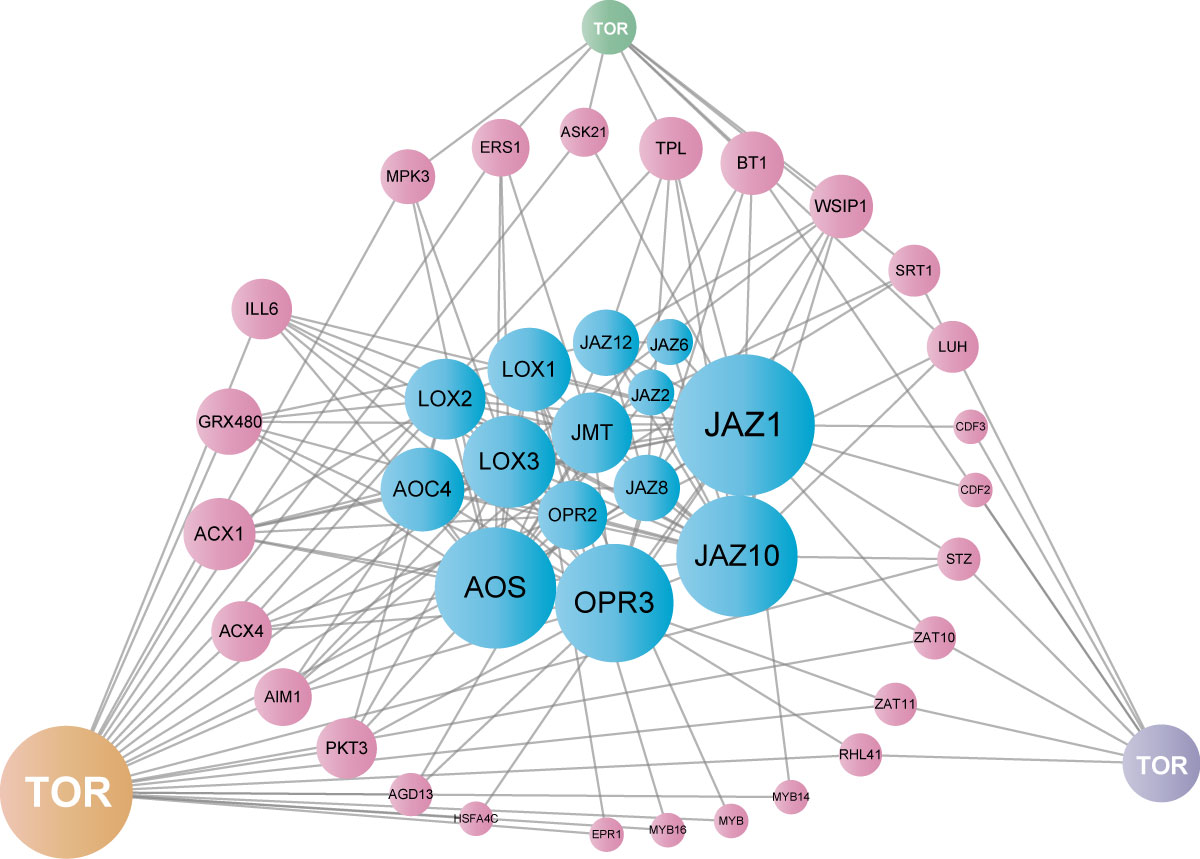


Supplemental Figure 4. Protein-protein interaction (PPI) network analysis of DEGs related to TOR and JA. Protein interaction relationship of targeted DEGs were constructed based on genome-wide PPI network of apple (GDDH13). The predicted protein associations were reference to the STRING database based on PPI from experimental evidences, textmining evidences, database evidences and coexpression evidences. The size of the node is the parameter of the connectivity of the network. Multiple copies of identical gene were illustrated as one node. The green circle TOR represented 5 sequences, including *MD05G1107800*, *MD00G1031100*, *MD10G1086500*, *MD10G1112000* and *MD15G1045400*. They coincided completely in PPI network. The orange circle TOR represented novel.4829, and the purple circle TOR represented novel.6269 in RNA-seq data.

Supplemental Table 1 Primer sequences used in this study.

| For qPCR | |
| --- | --- |
| Gene Name | Primer sequence (5’-3’) |
| *MDH* | F: CGTGATTGGGTACTTGGAAC  R:TGGCAAGTGACTGGGAATGA |
| *MdATG3a* | F: AAGGGGGCGGAGATGGTTC  R: GCACTTAGAGACGAGGTTATCGC |
| *MdATG3b* | F: AGGGAGATGGTTTTGAAACAGA  R: ACTTAGAGACGAGGTTATCGC |
| *MdATG4a* | F: GCCTCCAAGCTGGCAGATGAATC  R: CCACTATCACCCAACGCATCACTG |
| *MdATG5a* | F: GCAGGTCGTGTTCCAGTTC  R: CCTCCTCCTCCTTGTATCTCAA |
| *MdATG7a* | F: GCGGATATGAGCAACCTTGGC  R: ATCAATAGGCGCAACGACATCA |
| *MdATG7b* | F: ATCGGTAACAGGAGTAAGTCGG  R: TTTATCAAGCGCATGAAAGCCT |
| *MdATG8c* | F: GCGTTCAAGATGGAGCACCCTC  R: CAGCCCTTTCCACAACCACTGG |
| *MdATG8f* | F: TCGTAGACAATGTCCTCCCAGC  R: CCAAATGTGTTCTCGCCACTGT |
| *MdATG10* | F: TGGAACCAGCGAGTGGATGAAG  R: ACAACTGAGAGCCAAGACACCA |
| *MdATG11* | F: GAAGCGTTATTCACAACAACATCG  R: TTCCTCAAGTTCTCTTCCTTCACAA |
| *MdATG12* | F: ACAGTGCATTCTCGCCAAACCC  R: CCCCATGCCATGGAGCAAGC |
| *MdATG18a* | F: ATGATTCCAGGCTTGCCTGCTTTG  R: TGCAGCAAAGTTCCGTCGAGAGTA |
| For yeast two hybrid assay | |
| MD05G1107800-618N-BD | F: GGCCATGGAGGCCGAATTCGTCATGGTTCCCTTGTACCTTCTT  R: GCTGCAGGTCGACGGATCCAGCCACAGAAATTGCAAACTCTC |
| MD10G1086500-400N-BD | F: GGCCATGGAGGCCGAATTCATGCAAGGACTCCATCACCAAC  R: GCTGCAGGTCGACGGATCCGCCAAACTTTTGCAAAGATAGTAATTGGA |
| JAZ10-AD | F: CATGGAGGCCAGTGAATTCATGCCCAGAGCCACCGTTG  R: GCTCGAGCTCGATGGATCCTTAGGTTTGGCAGGCATATGGAGACC |
| JMT-AD | F: CATGGAGGCCAGTGAATTC ATGGAGGTGCTGCAAGTACTTCA  R: GCTCGAGCTCGATGGATCCTCAGTTCTTTCTAATGACCGACAAAACCA |
| NPR3-AD | F: CATGGAGGCCAGTGAATTC ATGGCTTATTCAGCTGAACCATCATCC  R: GCTCGAGCTCGATGGATCCTCACAATTTCCTAACCTTCTGATTTGTGCC |

Supplemental Table 2. FPKM values of DEGs related to photosynthesis, nitrogen, starch, anthocyanins, and plant hormone metabolism, protein degradation, autophagy, and SAGs in different treatments.


Note: Supplemental Table 2 is an inserted Excel table, since the DEGs were too much to be listed out. The excel table will be activated after double click. NC: Plants without any treatment; 0A: low N stress treatment (0.2 mM N); 1A and 10A: low N stress with 1 µM (1A) and 10 µM (10A) AZD8055 treatments.

Supplemental Table 3. The content of all metabolites of plant hormones detected by UPLC and MS/MS in different treatments.

Note: Supplemental Table 3 is an inserted Excel table, since the metabolites were too much to be listed out. The excel table will be activated after double click. NC: Plants without any treatment; 0A: low N stress treatment (0.2 mM N); 1A and 10A: low N stress with 1 µM (1A) and 10 µM (10A) AZD8055 treatments.
